# Supplementary material for: Dopamine neuronal loss contributes to memory and reward dysfunction in a model of Alzheimer's disease
Source: Nat Commun. 2017 Apr 3;8:14727. doi: 10.1038/ncomms14727 (PMC5382255; doi:10.1038/ncomms14727)
Supplement: Supplementary Information — Supplementary Figures [file ncomms14727-s1.pdf]

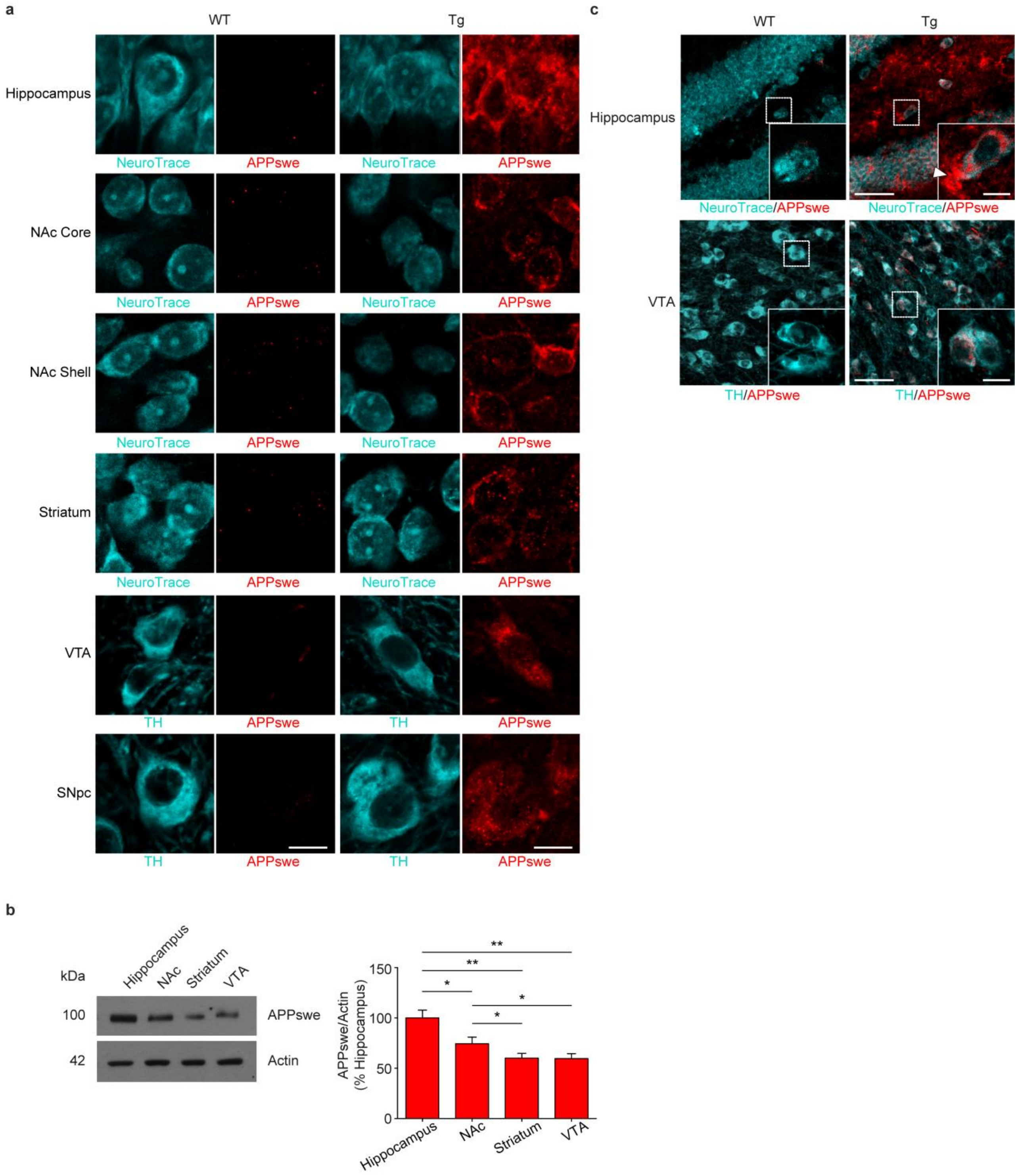

Supplementary Figure 1: Characterization of APPswe expression and Aβ-plaque deposition in the midbrain and midbrain projection areas in WT and Tg2576 mice. (**a**) Double-labelled confocal images for APPswe and NeuroTrace in coronal brain sections containing the hippocampus, striatum, NAc core and NAc shell and for APPswe and TH in the VTA and SNpc

from 6-month-old WT and Tg2576 mice (scale bar: 10 μm). APPswe is expressed in the cytoplasm of Tg2576 neurons in all the areas analyzed. Note the more intense staining in the hippocampus and NAc shell of Tg2576 mice in contrast to the light, diffuse intracellular staining in VTA and SNpc DAergic neurons (n = 3-5 mice per genotype per brain area; 4 sections per animal). (**b**) Representative immunoblots of full-lenght APPswe protein extracted from the indicated areas of 6-month-old Tg2576 mice. Actin was used as loading control. The bar graph represents densitometric quantification of changes in grey values of APPswe protein expression (n = 4 mice per brain area). APPswe protein is expressed more abundantly in the hippocampus and NAc (two-tailed unpaired *t*-test: hippocampus-NAc, $^{*}P = 0.043$; hippocampus-striatum, $^{**}P = 0.002$; hippocampus-VTA, $^{**}P = 0.002$; NAc-striatum, $^{*}P = 0.048$; NAc-VTA, $^{*}P = 0.038$). Data are mean ± s.e.m. (**c**) Double-labelling for APPswe and TH in the VTA (top) or NeuroTrace in the hippocampus dentate gyrus (bottom) in a coronal brain section of VTA and hippocampus from 11-month-old WT and Tg2576 mice (scale bar: 50 μm; inset: 10 μm). Hippocampal deposition of extracellular aggregates of APPswe (Aβ-plaques) is indicated by the arrowhead (n = 3 mice per genotype; 4 sections per animal).

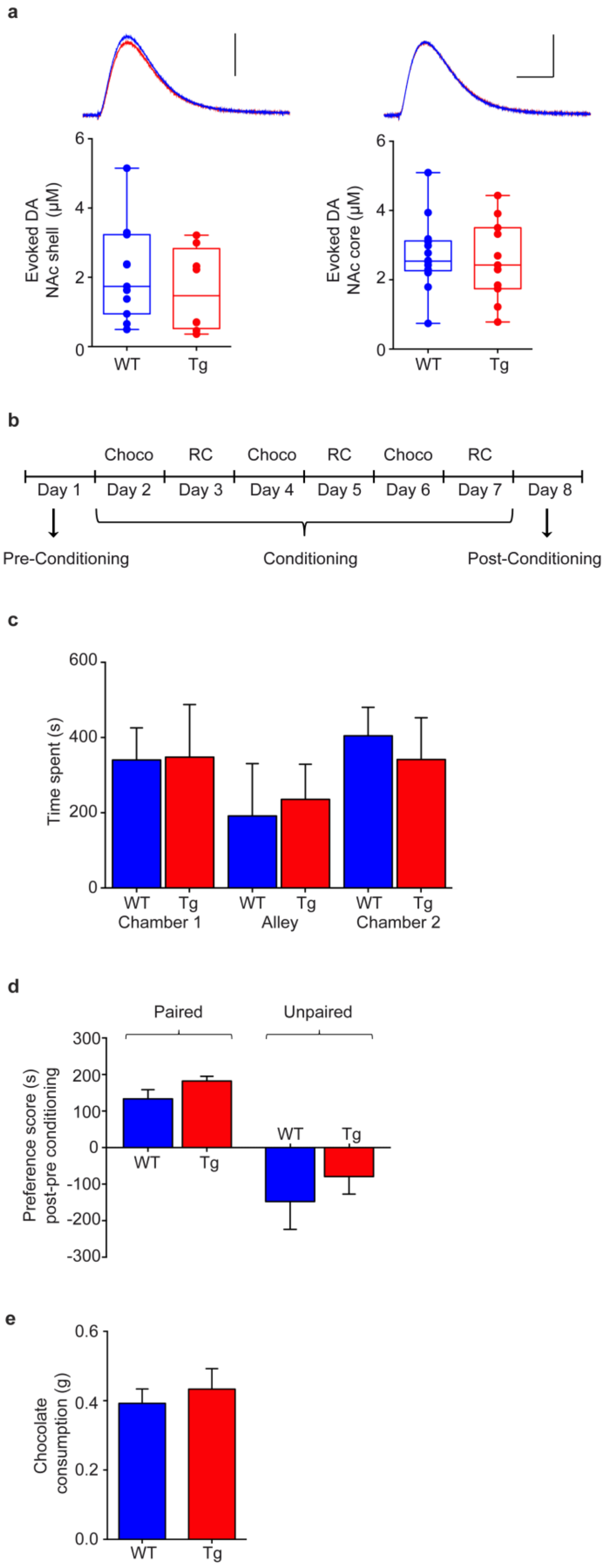
a
Evoked DA NAc shell (μM)
6
4
2
0
WT
Tg
Evoked DA NAc core (μM)
6
4
2
0
WT
Tg
b
Choco
RC
Choco
RC
Choco
RC
Day 1
Day 2
Day 3
Day 4
Day 5
Day 6
Day 7
Day 8
Pre-Conditioning
Conditioning
Post-Conditioning
c
Time spent (s)
600
400
200
0
WT
Tg
Chamber 1
WT
Tg
Alley
WT
Tg
Chamber 2
d
Paired
Unpaired
Preference score (s) post-pre conditioning
300
200
100
0
-100
-200
-300
WT
Tg
WT
Tg
e
Chocolate consumption (g)
0.6
0.4
0.2
0.0
WT
Tg

Supplementary Figure 2: Amperometric measurements of DA outflow in the NAc of 2-month-old mice and additional measurements of mesolimbic reward processing. (**a**) Evoked DA concentration in the NAc shell and core and example traces from 2-month-old WT and Tg2576 mice (scale bars: 50 pA, 250 ms), recorded with a carbon fiber electrode of equal calibration. In box-and-whisker plots, centre lines denote medians, edges represent upper and lower quartiles and whiskers show minimum and maximum values. Points are individual experiments (n = 11-14 slices from 3 WT mice, 8-11 slices from 3 Tg2576 mice)**.** (**b**) Schematic timeline of the experimental procedure during a CPP task. The task is divided in pre-conditioning, conditioning and post-conditioning sessions. During the conditioning session, mice are conditioned on alternate days with palatable food (chocolate; choco) or regular chow (RC). Testing is conducted during the post-conditioning session. (**c**) Pre-conditioning session of CPP in 6-month-old WT and Tg2576 mice (n = 5 each) showing no initial preference for either chambers ($F_{1,8}$ = 1.85; $P < 0.21$), no differences between genotypes ($F_{1,8}$ = 0.098; $P < 0.76$), nor significant interaction between genotype and chambers ($F_{1,8}$ = 0.10; $P < 0.75$). (**d**) Chocolate-induced place preference in 2-month-old WT and Tg2576 mice (WT: n = 4; Tg2576: n = 3) showing mean time spent in paired and unpaired chambers in post-conditioning session, minus the time spent in the same chambers during the pre-conditioning session. The repeated-measure ANOVA revealed no significant genotype effect ($F_{1,5}$ = 1.46; $P < 0.280$), a significant chamber effect ($F_{1,5}$ = 24.45; $P < 0.004$) and no significant genotype x chambers interaction ($F_{1,5}$ = 0.03; $P < 0.860$). (**e**) Chocolate consumption in 2-month-old WT and Tg2576 mice (WT: n = 4; Tg2576: n = 3) during conditioning sessions. Two-month-old WT and Tg2576 mice show similar preference for the chocolate-paired chamber and consume equal amounts of chocolate. Data in **c-e** are expressed as mean ± s.e.m.

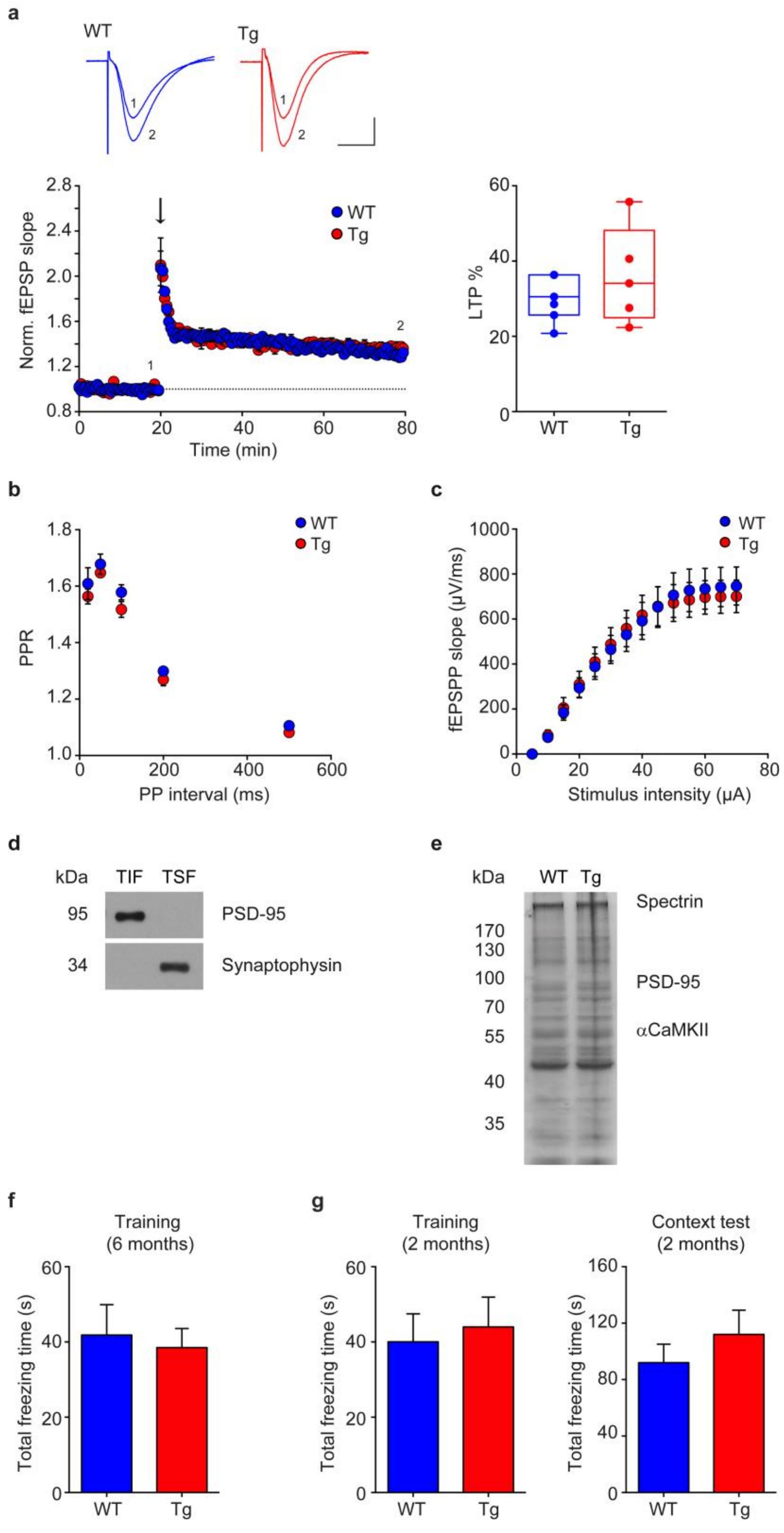

Supplementary Figure 3: Two-month-old Tg2576 mice do not show deficits in synaptic plasticity and contextual fear conditioning performance. (**a**) The plot shows normalized CA3-to-CA1 fEPSP mean slope (± s.e.m. every 2 min) recorded from the hippocampal CA1 dendritic region from 2-month-old mice. The arrow indicates when a high frequency conditioning train was

delivered, after a 20 min baseline. The traces (scale bar: 200 μV, 10 ms) show superimposed fEPSPs recorded during baseline (1) and 1 hour after the train (2). The box-and-whisker plot indicates the degree of potentiation, as fEPSP slope increase from baseline 55-60 min after the train. At this age the LTP is unchanged in Tg2576 mice (WT: n = 7 slices from 3 mice; Tg2576: n = 5 slices from 3 mice). (**b**) PPR of the CA1 fEPSP slope evoked at half-maximal Schaffer collateral stimulation intensity, at increasing paired-pulse intervals (20, 50, 100, 200 and 500 ms) from 6-month-old WT (n = 4) and Tg2576 (n = 5) mice. (**c**) Input/output curves of the CA1 fEPSP slope at stimulations of increasing intensity, from 6-month-old WT (n = 4) and Tg2576 (n = 5) mice. (**d,e**) Immunoblot analysis of PSD-95 and synaptophysin demonstrating the purity of the PSD preparation: (**d**) Representative immunoblots of the PSD fraction (Triton Insoluble Fraction, TIF) and of the fraction containing synaptic cytosolic components (Triton Soluble Fraction, TSF); (**e**) PSD protein preparations from 6-month-old mice were separated by SDS-PAGE and stained with Coomassie brilliant blue. No major differences are seen between genotypes at this level of resolution. (**f**) Total freezing time during the CFC training session in 6-month-old mice (n = 6 mice per genotype). (**g**) Total freezing time during the CFC training (left) and context test (right) in 2-month-old mice (n = 5 mice each). Two-month-old Tg2576 mice do not show deficits in memory performance. Excluding the box-and-whisker plot in **a**, all data are expressed as mean ± s.e.m.

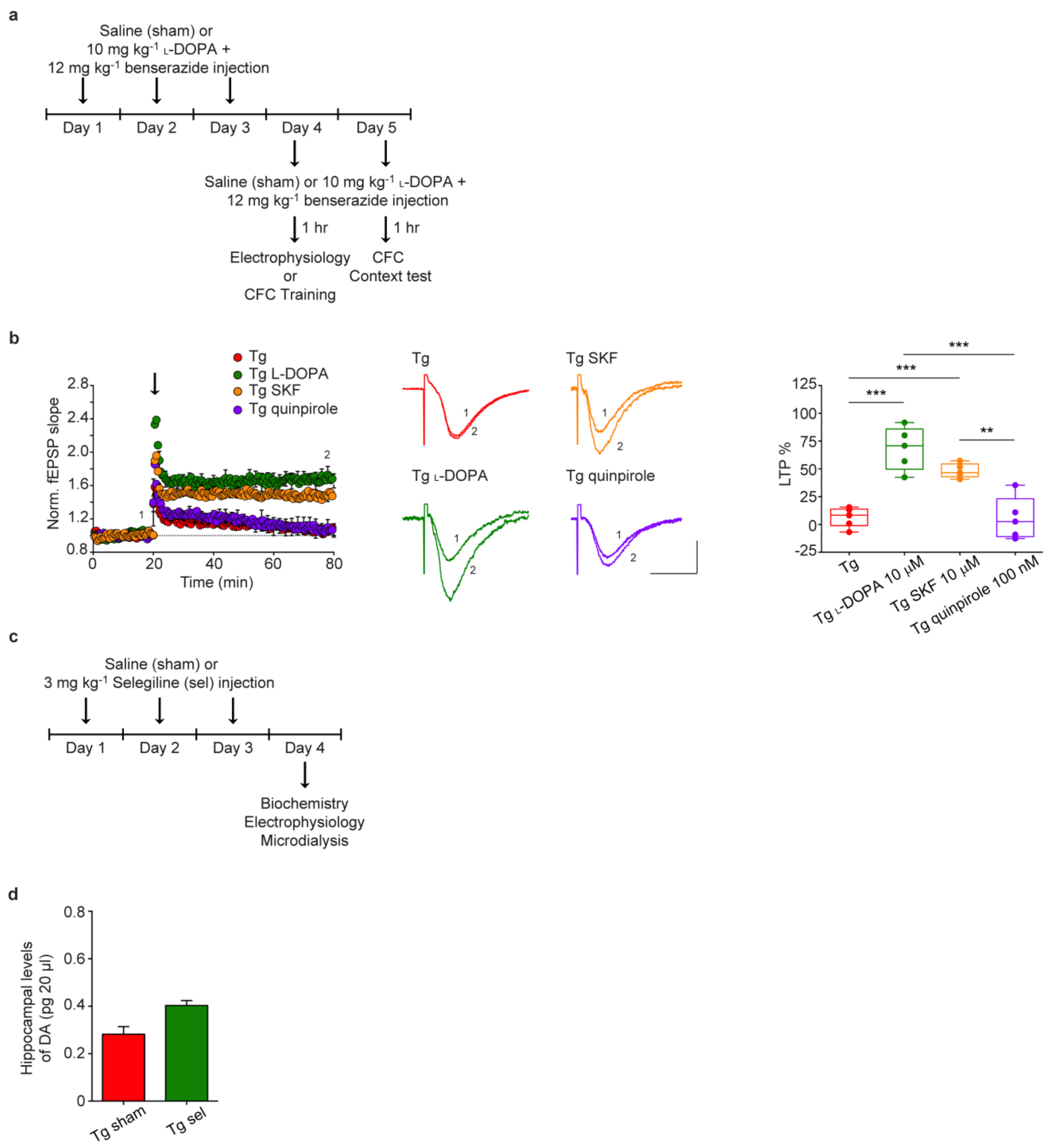

Supplementary Figure 4: L-DOPA and selegiline experimental timeline of treatment and additional measurements. (**a**) Experimental procedure for the sub-chronic L-DOPA (10 mg kg^{-1} in benserazide 12 mg kg^{-1}) and saline treatment in 6-month-old WT and Tg2576 mice. Single intraperitoneal injections (5 µl per animal weight in grams) were done for 4 consecutive days and mice were used for LTP recordings or the CFC training session 1 hour after the last injection. For the CFC context session, mice were also injected on day 5, 1 hour before testing. (**b**) The plot

shows the effect of acute, bath-applied administration of L-DOPA (10 µM), SKF38393 (SKF, 10 µM) and quinpirole (100 nM) on the CA3-to-CA1 LTP (mean slope ± s.e.m. every 2 min) recorded from the hippocampal CA1 dendritic region in 6-month-old Tg2576 mice. The arrow indicates when a high frequency conditioning train was delivered. The traces (scale bar: 100 µV, 10 ms) are superimposed fEPSPs recorded during baseline (1) and 1 hour after the train (2) and the box-and-whisker plot indicates the degree of potentiation, as fEPSP slope increase from baseline 55-60 min after the train. Both L-DOPA and the D1 selective agonist SKF rescue LTP in Tg2576 mice (n = 6 Tg slices, 5 Tg L-DOPA, 5 Tg SKF, 5 Tg quinpirole; one-way ANOVA $F_{3,17} = 25.05$, $P < 1.00 \times 10^{-4}$; Tg-Tg L-DOPA ***$P < 0.001$, Tg-Tg SKF ***$P < 0.001$, Tg L-DOPA-Tg quinpirole ***$P < 0.001$, Tg SKF-Tg quinpirole **$P < 0.010$ with Bonferroni's post-hoc test). (**c**) Experimental procedure for the selegiline (3 mg kg^{-1}) and saline sub-chronic treatment in 6-month-old mice used for electrophysiology, biochemistry and *in-vivo* microdialysis experiments. Single subcutaneous injections (5 µl per animal weight in grams) were done for three consecutive days and animals were sacrificed on the fourth day. For behavioural tests, see treatment schemes in **Supplementary Fig. 5**. (**d**) *In-vivo* microdialysis measurements of DA outflow in the hippocampus of 6-month-old sham and sel-treated Tg2576 mice (n = 5 mice each; one-way ANOVA: $F_{1,8} = 10.134$, *$P = 0.013$). All data are expressed as mean ± s.e.m.

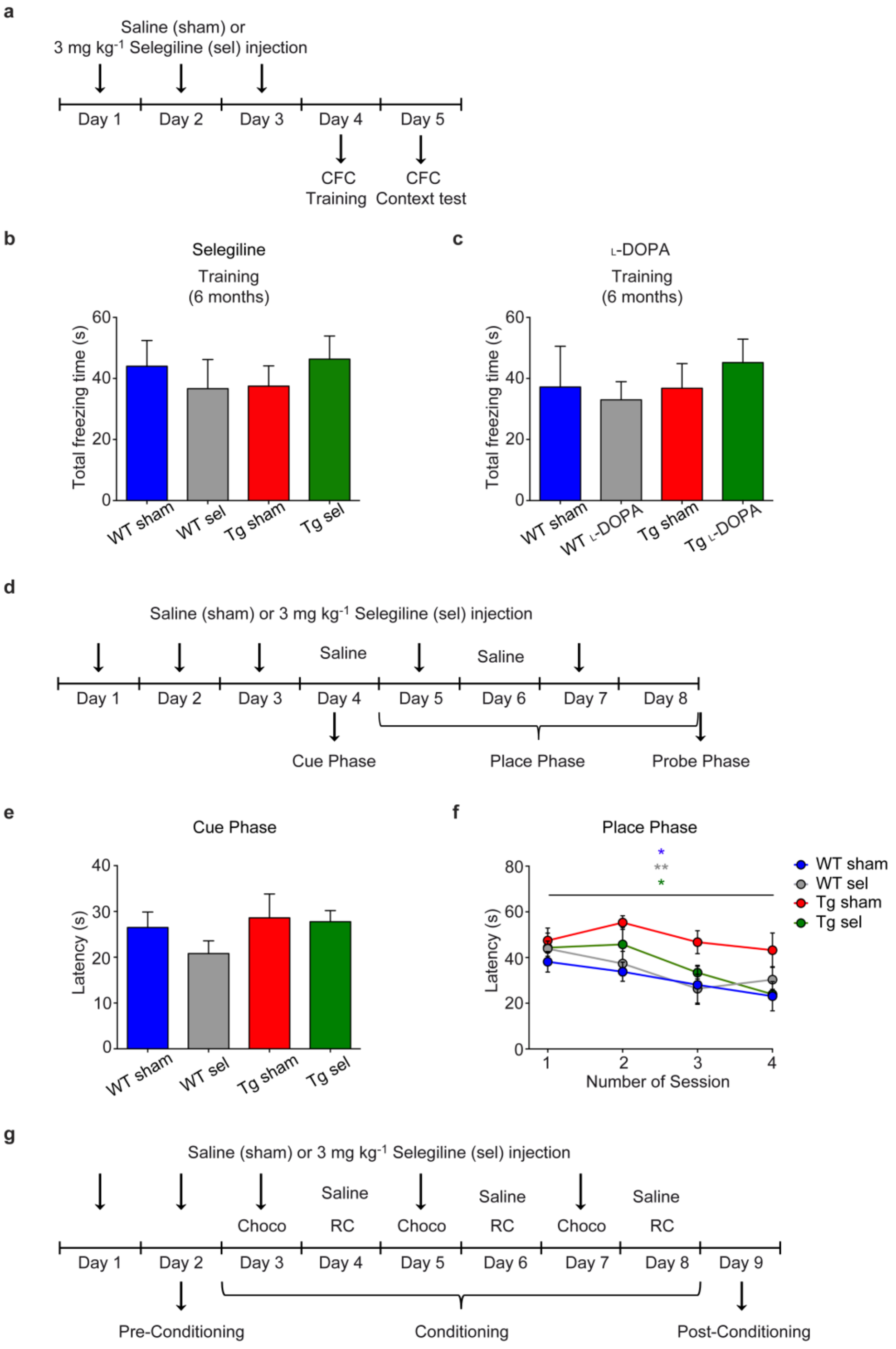

Supplementary Figure 5: Behavioural experiments following sub-chronic selegiline or L-DOPA treatment. (**a**) Experimental procedure for the selegiline (3 mg kg^{-1}; 5 µl per animal weight in grams) and saline sub-chronic treatment in 6-month-old mice used for CFC. (**b**,**c**) Total freezing time during the CFC training session in 6-month-old sham and sel-treated WT and Tg2576 mice (**b**; 6 mice per group) and in sham- and L-DOPA-treated mice (**c**; 5 mice per group).

(**d**) Experimental procedure for the selegiline (3 mg kg^{-1}) and saline sub-chronic treatment in 6-month-old mice tested with the Morris water maze (MWM) test. (**e**,**f**) Time spent to reach the platform during the Cue phase (**e**) and Place phase (**f**) of the MWM test. No significantly different latencies in reaching the visible platform were found among groups (n = 5 mice per group) during Cue phase. During Place phase, Tg2576 sham mice exhibited significantly longer latencies in finding the platform in comparison to the remaining groups, indicating an impaired spatial learning that was prevented by selegiline treatment (two-tailed paired *t*-test on mean latencies of session 1 *versus* session 4: WT sham, *P = 0.048; WT sel, **P = 0.009; Tg sham, P = 0.511; Tg sel, *P = 0.024). (**g**) Experimental timeline for the selegiline (3 mg kg^{-1}; 5 μl per animal weight in grams) and saline sub-chronic treatment in 6-month-old mice used for the CPP task. The task was divided in pre-conditioning, conditioning and post-conditioning sessions and during the conditioning session mice were conditioned on alternate days with chocolate (choco) or regular chow (RC). Testing was conducted during the post-conditioning session. All data shown represent mean values ± s.e.m.

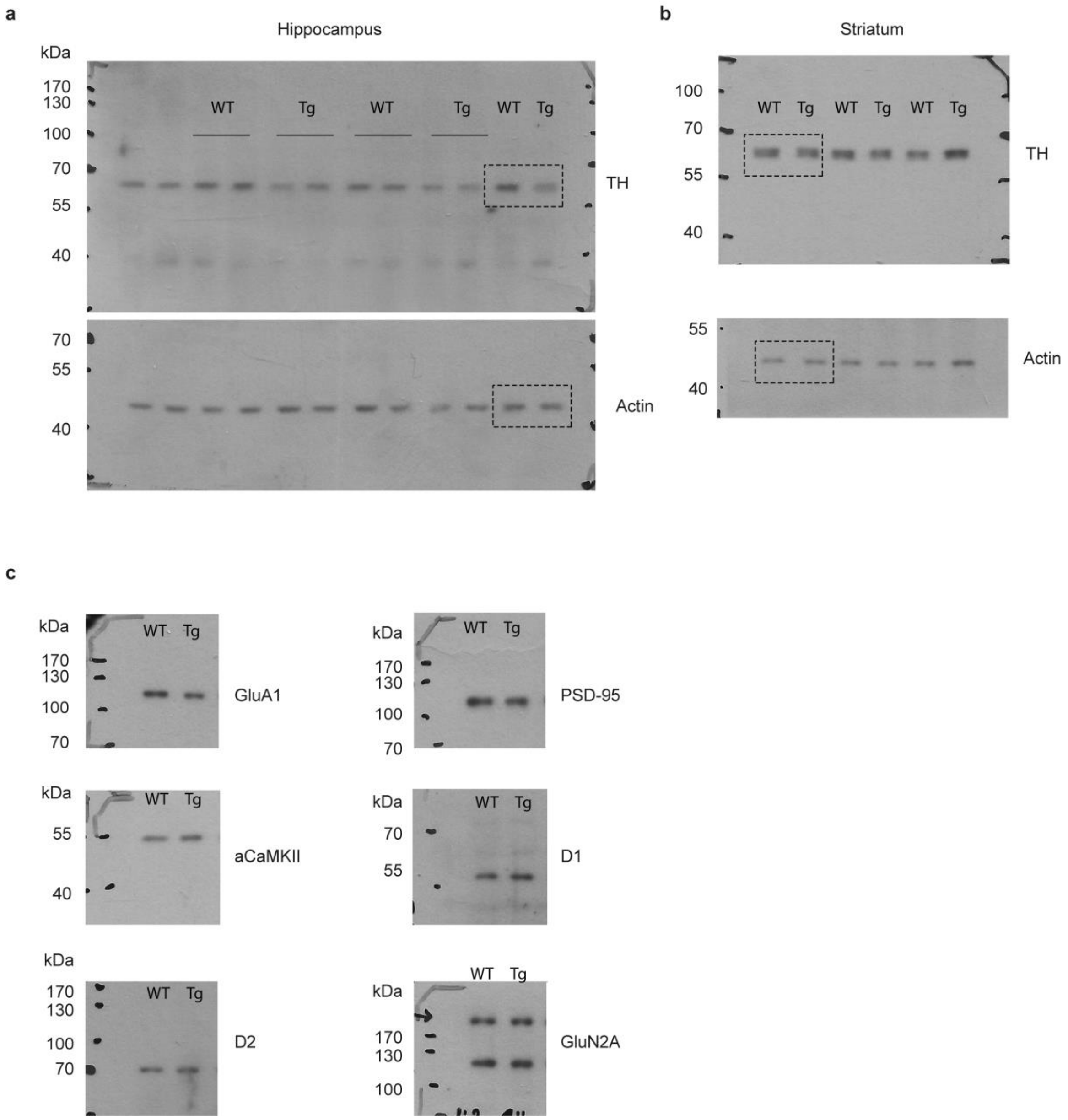

Supplementary Figure 6: Original scans used in the main text figures. (**a**) Blots from Figure 4e. (**b**) Blots from Figure 4g. (**c**) Blots from Figure 4i.

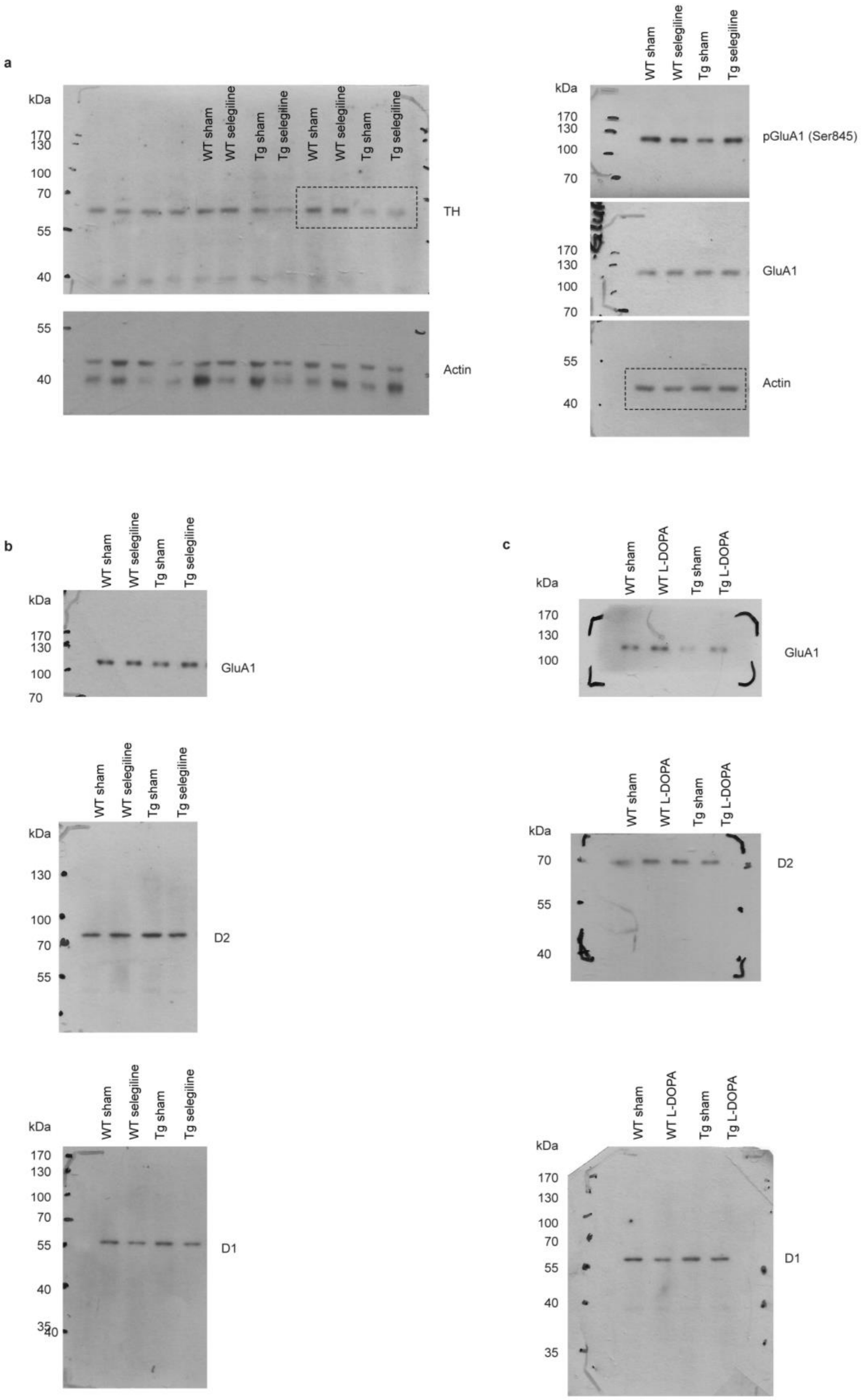

Supplementary Figure 7: Original scans used in the main text figures. (**a**) Blots from Figure 6a. (**b**) Blots from Figure 6b. (**c**) Blots from Figure 6c.

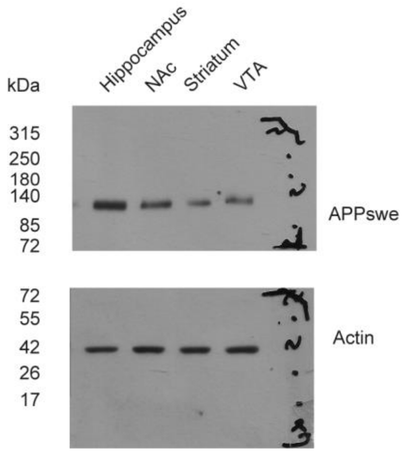

Supplementary Figure 8: Original scans used in Supplementary Figure 1.
